# Role of potassium and calcium channels in sevoflurane-mediated vasodilation in the foeto-placental circulation

**DOI:** 10.1186/1471-2253-9-4

**Published:** 2009-06-10

**Authors:** James Jarman, Chrisen H Maharaj, Brendan D Higgins, Rachel F Farragher, Christopher D Laffey, Noel M Flynn, John G Laffey

**Affiliations:** 1 Department of Anaesthesia, Clinical Sciences Institute, National University of Ireland, Galway Ireland; 2 National Centre for Biomedical Engineering Sciences, National University of Ireland, Galway, Ireland; 3 Public Analysts Laboratory, Newcastle, Galway, Ireland

## Abstract

**Background:**

Sevoflurane has been demonstrated to vasodilate the foeto-placental vasculature. We aimed to determine the contribution of modulation of potassium and calcium channel function to the vasodilatory effect of sevoflurane in isolated human chorionic plate arterial rings.

**Methods:**

Quadruplicate *ex vivo *human chorionic plate arterial rings were used in all studies. ***Series 1 and 2 ***examined the role of the K^+ ^channel in sevoflurane-mediated vasodilation. Separate experiments examined whether tetraethylammonium, which blocks large conductance calcium activated K^+ ^(K_Ca++_) channels (***Series 1A+B***) or glibenclamide, which blocks the ATP sensitive K^+ ^(K_ATP_) channel (***Series 2***), modulated sevoflurane-mediated vasodilation. ***Series 3 – 5 ***examined the role of the Ca^++ ^channel in sevoflurane induced vasodilation. Separate experiments examined whether verapamil, which blocks the sarcolemmal voltage-operated Ca^++ ^channel (***Series 3***), SK&F 96365 an inhibitor of sarcolemmal voltage-independent Ca^++ ^channels (***Series 4A+B***), or ryanodine an inhibitor of the sarcoplasmic reticulum Ca^++ ^channel (***Series 5A+B***), modulated sevoflurane-mediated vasodilation.

**Results:**

Sevoflurane produced dose dependent vasodilatation of chorionic plate arterial rings in all studies. Prior blockade of the K_Ca++ _and K_ATP _channels augmented the vasodilator effects of sevoflurane. Furthermore, exposure of rings to sevoflurane in advance of TEA occluded the effects of TEA. Taken together, these findings suggest that sevoflurane blocks K^+ ^channels. Blockade of the voltage-operated Ca^++^channels inhibited the vasodilator effects of sevoflurane. In contrast, blockade of the voltage-independent and sarcoplasmic reticulum Ca^++^channels did not alter sevoflurane vasodilation.

**Conclusion:**

Sevoflurane appears to block chorionic arterial K_Ca++ _and K_ATP _channels. Sevoflurane also blocks voltage-operated calcium channels, and exerts a net vasodilatory effect in the *in vitro *foeto-placental circulation.

## Background

Sevoflurane has gained widespread acceptance in obstetric anaesthesia [1-3], and has an excellent clinical profile in this population [2,3]. We have recently demonstrated that sevoflurane vasodilates the foeto-placental vasculature *in vitro*, in part by a mechanism that is mediated *via *inhibition of lipoxygenase [4]. However, the potential for sevoflurane to cause vasodilation *via *modulation of calcium and potassium ion channel function in this circulation remains to be determined.

The potential for volatile anaesthetic agents to modulate vascular tone *via *alterations in calcium and potassium ion channel function in other circulations is clear. Halothane has been demonstrated to vasodilate conducting and resistance coronary arteries *via *activation of the K_ATP _channel [5]. Sevoflurane-mediated activation of potassium ion (K^+^) channels would increase potassium conductance, causing myocyte hyperpolarisation and vasodilation [6]. Two types of K^+ ^channels of functional importance in the vasculature are the Ca^++^-activated K^+ ^(K_Ca++_) channels and the ATP-sensitive K^+ ^(K_ATP_) channels [6]. The effects of sevoflurane on to K^+ ^channels appear to be depend on channel subtype. Sevoflurane has been demonstrated to selectively increase coronary collateral blood flow *via *mechanisms that appear to be independent of K_ATP _channel activation [7], but which involve activation of K_Ca++ _channels [8].

Sevoflurane could also cause vasodilation *via *inhibition of one or more calcium (Ca^++^) channels. At least three distinct types of Ca^++ ^channels are of importance in the vasculature: the sarcolemmal voltage-operated Ca^++ ^channel (VOCC) [9]; sarcolemmal voltage-independent Ca^++ ^channels (VICC) [9,10]; and sarcoplasmic reticulum calcium channel. Of interest, halothane has been demonstrated to activate VICC channel activity, while sevoflurane did not have any effect on these channels, in the rat aorta [9].

We therefore hypothesized that sevoflurane would vasodilate the foeto-placental circulation by a mechanism involving alterations in the activity of potassium and/or calcium channels. Specifically, we hypothesized that sevoflurane activates K^+ ^channels, causing myocyte hyperpolarisation and vasodilation. Our second hypothesis was that sevoflurane inhibits Ca^++ ^channels, thereby reducing intracellular Ca^++ ^concentrations, and thus directly reduces vessel tension.

## Methods

Following ethical committee approval, and written informed patient consent, term placentae were obtained after both vaginal and caesarean delivery under neuraxial anaesthesia from healthy parturients. Exclusion criteria included pre-existing hypertension, intra-uterine growth retardation, pre-eclampsia, multiple pregnancies, and patients with pregnancy induced hypertension, hepatitis and HIV infection.

**Figure 1:**
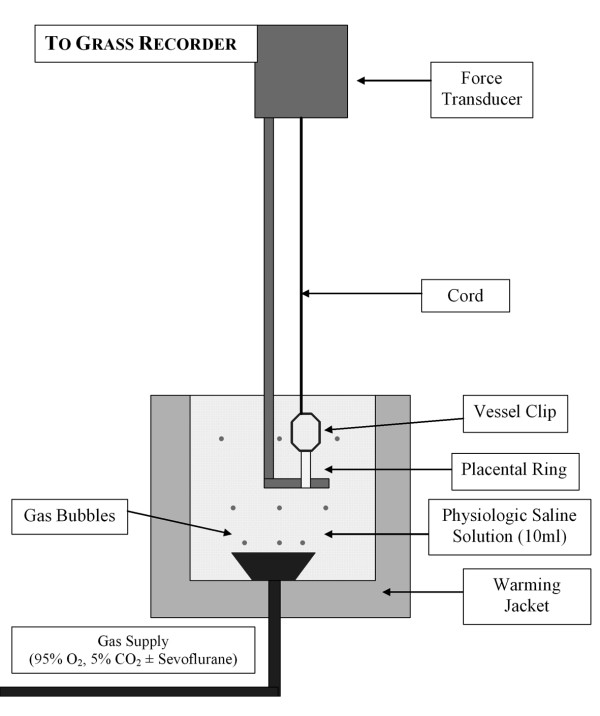
**Schematic representation of the ex vivo incubation model system used in the isolated vessel experiments**.

**Figure 2:**
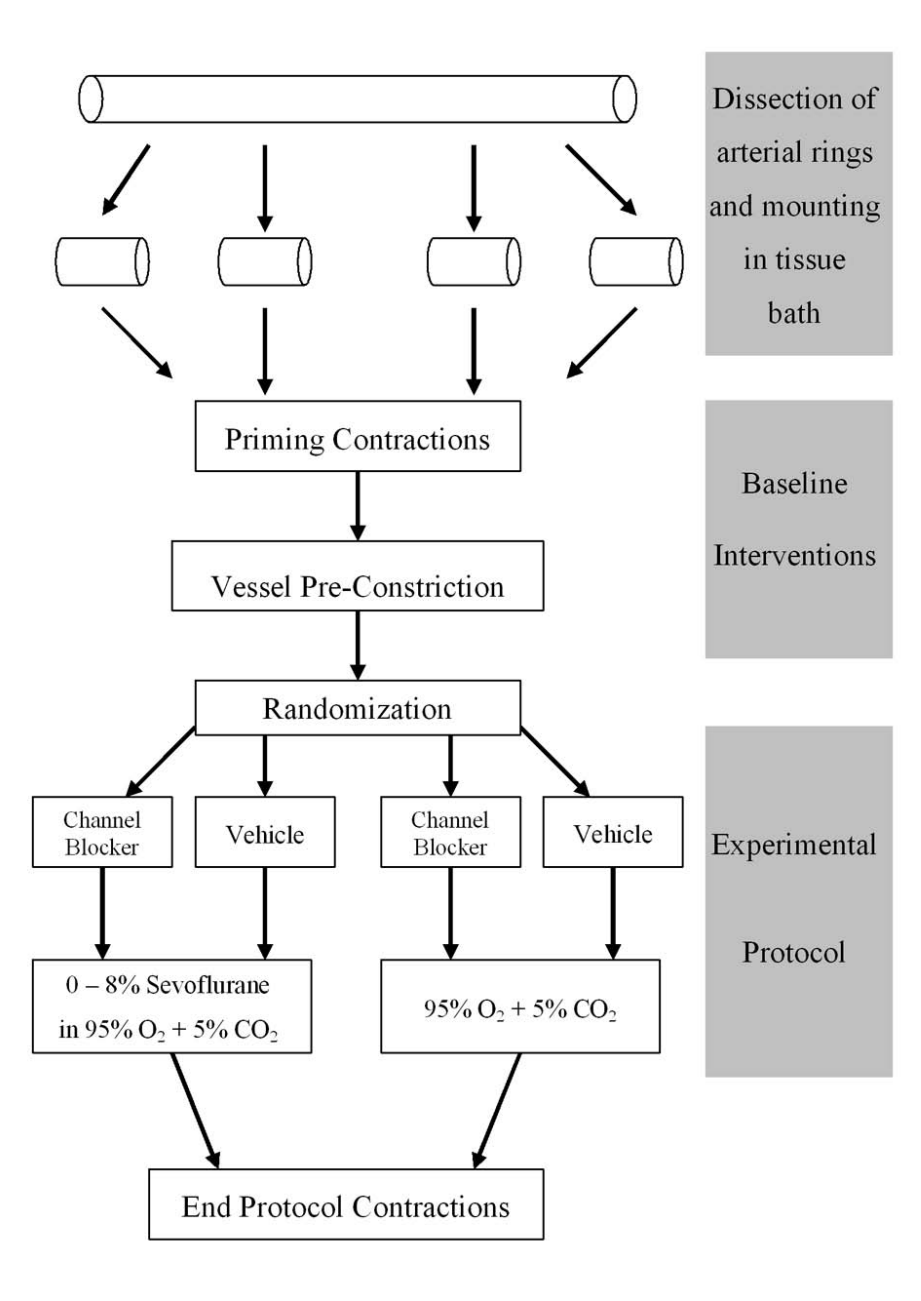
**Schematic representation of the experimental design used in each experimental series, with the exception of series 1B**.

All studies followed a randomised, controlled, paired design. Samples of the second-order chorionic plate arteries were taken within 120 minutes of delivery and placed directly into ice-cold pyrogen-free physiologic saline solution. Four chorionic rings, each 3 mm in length, from a single artery from each placenta, were isolated, mounted in PSS at 37°C, and equilibrated with 95% O_2 _– 5% CO_2 _in tissue baths (10 ml capacity) as previously described [4,11] (Figure 1). Samples of the solution were intermittently analyzed for P_O2_, P_CO2 _and pH. P_CO2 _was maintained in the range 3.5 – 4.5 kPa, and pH was maintained in the range 7.35 – 7.45 in all experiments. Rings were threaded onto a horizontally fixed platinum surgical wire (300 μm diameter). A second hook, connected to an isometric force transducer, was then passed through the lumen of the ring. Isometric tension was recorded as a function of time using a transducer system (Grass FT03, Quincy, MA) (Figure 2).

### Baseline Interventions

Following a 60 minute equilibration period, an optimal pre-tension of 2.00 g for each chorionic plate arterial ring was utilised [4]. Priming contractions were induced by exposing the rings three times to 80 mM KCl solution (iso-osmotically substituted for NaCl), and pre-tension re-established at the end of each 5 minute exposure by rinsing with physiologic saline solution (Figure 2). The rings were then submaximally pre-constricted with the thromboxane analog U46619 (9,11-dideoxy-11α, 9α-epoxymethanoprostagladin F2α, 5 × 10-6 M). Once a stable plateau contractile response was obtained, the rings were allowed to remain at the plateau tension for 30 minutes.

### K^+ ^Channel Experimental Series

Each experimental series followed a similar overall design (Figure 2). **Series 1 **examined the role of the calcium activated K^+ ^(K_Ca++_) channel in mediating the vasodilatory effect of sevoflurane. Tetraethylammonium (TEA), which at lower doses (1 – 3 × 10^-2 ^M), blocks large conductance calcium activated K^+ ^(K_Ca++_) channels [12-14] was utilised in these experiments. ***Series 1A ***determined whether prior incubation with TEA modulated the vasodilation produced by sevoflurane. Following U46619 pre-constriction, two rings from each placenta were randomly assigned to receive sevoflurane in the perfusing gas, in 2% increments sequentially attained at 30 minute intervals, with the remaining two rings serving as controls (Figure 2). TEA (1 × 10^-2 ^M) was first added to one sevoflurane exposed bath and one control bath, and allowed to equilibrate for 20 minutes, and vehicle added to the remaining baths. This resulted in four groups: (1) control with vehicle (Control); (2) control with TEA (TEA); (3) sevoflurane with vehicle (SEVO); (4) sevoflurane with TEA (SEVO+TEA). Increasing sevoflurane perfusate concentrations from 2% – 8% were then sequentially attained at 30 minute intervals.

***Series 1B ***determined the effect of prior incubation of the rings with sevoflurane on the vasoactive effect of TEA. Following U46619 pre-constriction and the attainment of a plateau contraction, two rings were randomly assigned to receive 8% sevoflurane in the perfusing gas (95% O_2 _– 5% CO_2_) or perfusing gas alone. After 60 minutes, TEA (1 × 10^-2 ^M) was added to one sevoflurane exposed bath and one control bath, and vehicle added to the remaining baths, and the effects measured. ***Series 2 ***examined the role of the ATP sensitive K^+ ^channel (K_ATP_) channel in mediating the vasodilatory effect of sevoflurane. Glibenclamide, which at lower doses (1 × 10^-5 ^M) specifically blocks the K_ATP _channel [15,16], was utilised in these experiments. Following U46619 pre-constriction, and the addition of 1 × 10^-5 ^M glibenclamide or vehicle, rings were exposed to sevoflurane or vehicle gas alone as described for series 1A.

### Ca^++ ^Channel Experimental Series

***Series 3 ***examined the role of the sarcolemmal voltage-operated Ca^++ ^channel (VOCC). Following U46619 pre-constriction, verapamil 1 × 10^-6 ^M, which specifically blocks the VOCC channel [9,17,18] or vehicle was added, and the rings exposed to sevoflurane or perfusing gas alone as described for series 1A. ***Series 4 ***examined the role of the sarcolemmal voltage-independent Ca^++ ^channels (VICC) [9,10]. SK&F 96365, which at concentrations in the 10^-4 ^– 10^-6 ^M range specifically blocks the VICC channel [9,10,19], was utilised in these experiments. ***Series 4A ***examined whether 3 × 10^-5 ^M SK&F 96365 blocked sevoflurane-mediated vasodilation. ***Series 4B ***examined the effect of higher concentrations of SK&F 96365 (1 × 10^-4 ^M). **Series 5 **examined the role of the sarcoplasmic reticulum Ca^++ ^channel [9,10]. Ryanodine, which at concentrations of up to 10^-4 ^M specifically inhibits sarcoplasmic reticulum calcium release [20,21] was utilised in these experiments. ***Series 5A ***examined the effect of 5 × 10^-6 ^M ryanodine, while ***Series 5B ***determined whether 5 × 10^-5 ^M ryanodine attenuated the vasodilation produced by sevoflurane.

### End protocol contractility assessment

At the end of each experiment, sevoflurane was discontinued, and baseline pre-tension re-established. The contractile response to 80 mM KCl was then reassessed and data was excluded from the analysis if the maximum final KCl-induced contraction in control rings was less than 90% of the initial KCl response.

### Determination of bath sevoflurane concentrations

The sevoflurane was introduced into the tissue baths in the perfusing gas *via *a sevoflurane vapouriser (Abbott Inc., Dublin, Ireland). The vapour content of anaesthetic agent in the carrier gas was continuously measured by means of an in-line agent monitor (Datex Capnomac Ultima^®^, Helsinki, Finland). The perfusing gas was continuously sampled downstream from the vapouriser, at a point just proximal to the entry of the gas into the tissue baths. Volatile anaesthetic concentrations increased within 10 minutes to within 10% of the value dialled on the vapouriser in all experiments. In addition, in a separate series of experiments, the bath concentrations of sevoflurane were verified using gas chromatography-mass spectrometry, for sevoflurane concentrations in the perfused gas of 0 – 8%, as previously described [4].

### Chemicals

U46619, tetraethylammonium (TEA)α, glibenclamide, verapamil, SK & F 96365, sodium nitroprusside, and all salts were all purchased from Sigma Aldrich (Poole, Dorset, U.K.). Ryanodine was purchased from BIOMOL International L.P. (Exeter, Devon, U.K.). Sevoflurane was purchased from Abbott Inc. (Dublin, Ireland).

### Statistical Analysis

Data are presented as means ± SD with vasodilation by sevoflurane expressed as a percentage of the submaximal contraction elicited with U46619. The EC_50r _value, which represents the concentration of sevoflurane required dilate the pre-constricted rings by 50%, was calculated for each ring, in each experimental series. Firstly, the slope and intercept of the line connecting the sevoflurane values immediately below and above 50% was determined. The EC_50r _value is calculated by the following formula: EC_50r _= 10^X ^where X = (50 – Intercept)/Slope. Comparison between control and test rings was made using two way repeated measures analysis of variance, with group as a between subjects factor and percentage sevoflurane as a repeated measures factor. Between group analyses were restricted to comparisons relevant to our *a priori *hypotheses, and were made using Student's t testing with corrections for multiple comparisons. The null hypothesis was rejected for p < 0.05.

## Results

Chorionic plate arterial rings were obtained from 80 placentae from women ((median age, 26 years [range, 18–43 years]; median parity, 1 [range, 0–4]) following uncomplicated full term (median gestation 40 wks [range 38–41]) gestation, for these studies. Stable and comparable gas tensions were maintained throughout all experiments, and comparable baseline levels of contractile responses were observed in all series. Post-intervention responses to potassium chloride were not different compared to baseline for any rings studied, and therefore no rings were excluded.

### Sevoflurane and the K+ Channel

**Figure 3:**
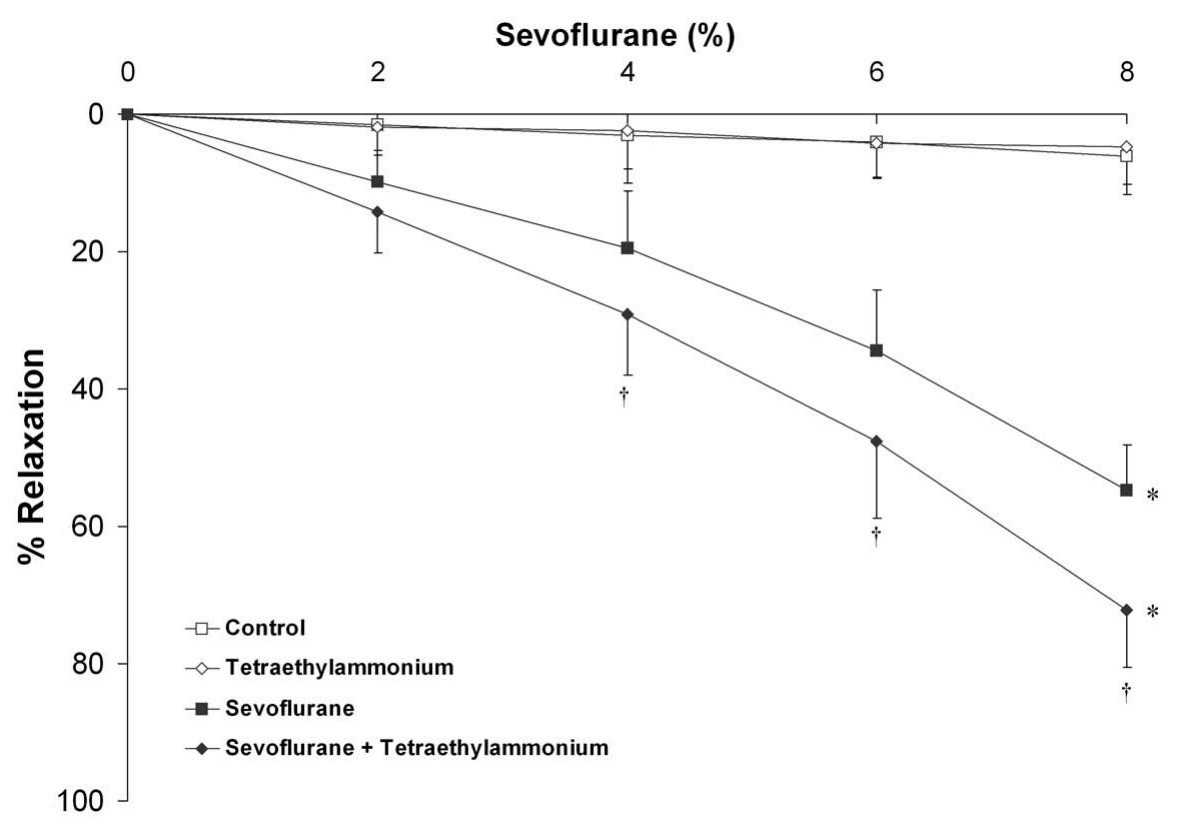
**Blockade of K_Ca++ _channels enhances the vasodilatory effect of sevoflurane**. Sevoflurane produced significant dose dependent vasodilation of chorionic plate arterial rings compared to control conditions. Prior blockade of K_Ca++ _channels by incubation in 1 × 10^-2 ^M tetraethylammonium (n = 10 rings per group) enhanced the vasodilatory effect of sevoflurane. *P < 0.05 compared to rings exposed to control conditions. (Two way RM-ANOVA). † P < 0.05 compared to rings exposed to sevoflurane plus vehicle at graded sevoflurane concentrations (post hoc between group t test).

**Figure 4:**
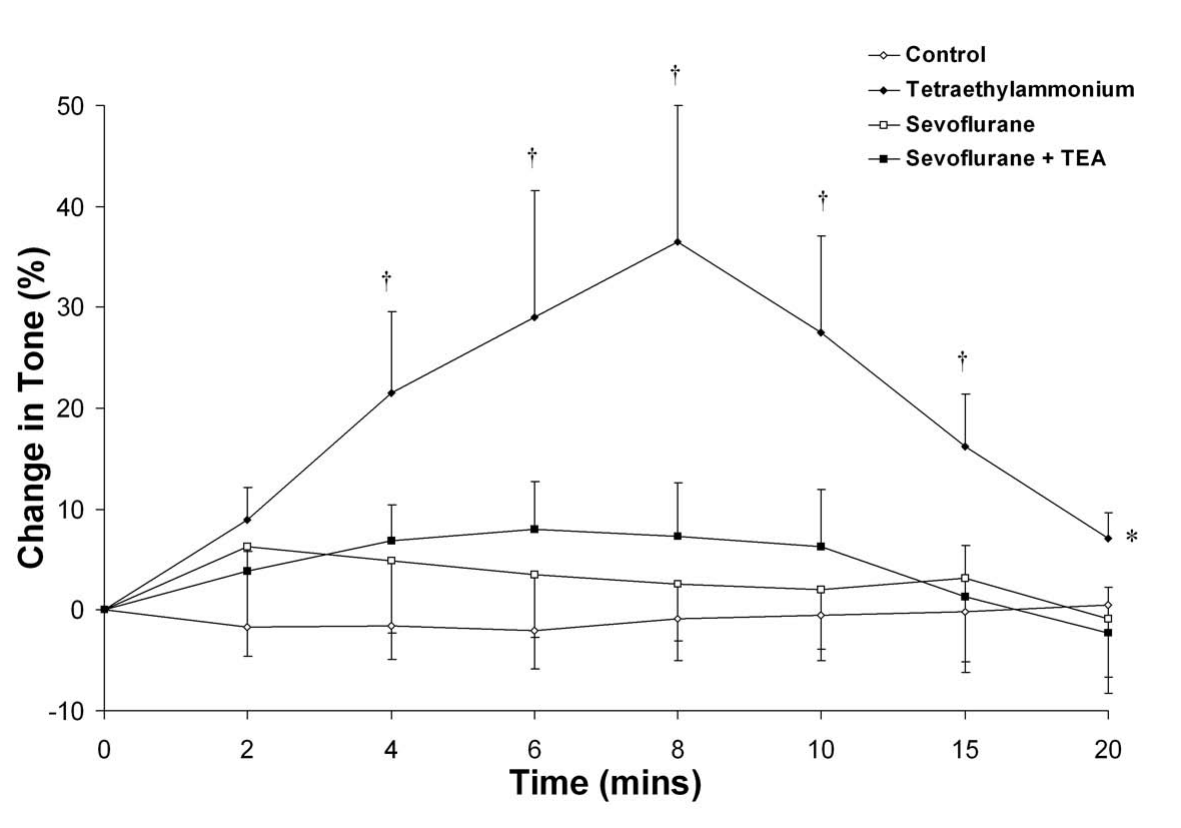
**Prior exposure of chorionic rings to sevoflurane blocks the effect of tetraethylammonium**. Exposure of rings to 1 × 10^-2 ^M tetraethylammonium transiently enhances resting tone. This effect is abolished following prior incubation in sevoflurane, demonstrating that sevoflurane blocks K_Ca++ _channels (n = 10 rings per group). *P < 0.05 compared to control (Two way RM-ANOVA). † P < 0.05 compared to control at each timepoint (post hoc between group t test).

In ***Series 1A***, blockade of the calcium activated K^+ ^(K_Ca++_) channel (n = 10 rings per group) with 1 × 10^-2 ^M TEA significantly increased sevoflurane-mediated vasodilation compared to sevoflurane alone (Figure 3). TEA pre-treatment significantly decreased the EC_50r _for sevoflurane (6.2 ± 0.8% versus 7.5 ± 0.7%, P < 0.01). In ***series 1B ***(n = 10 rings per group), 1 × 10^-2 ^M TEA caused marked but transient vasoconstriction, with a maximal contraction of 36.5 ± 3.1% attained 8 minutes following TEA addition (Figure 4). Prior incubation of rings with sevoflurane abolished the vasoconstrictor effect of TEA. This suggests that sevoflurane directly blocks the K_Ca++ _channel, thereby occluding the effect of TEA.

**Figure 5:**
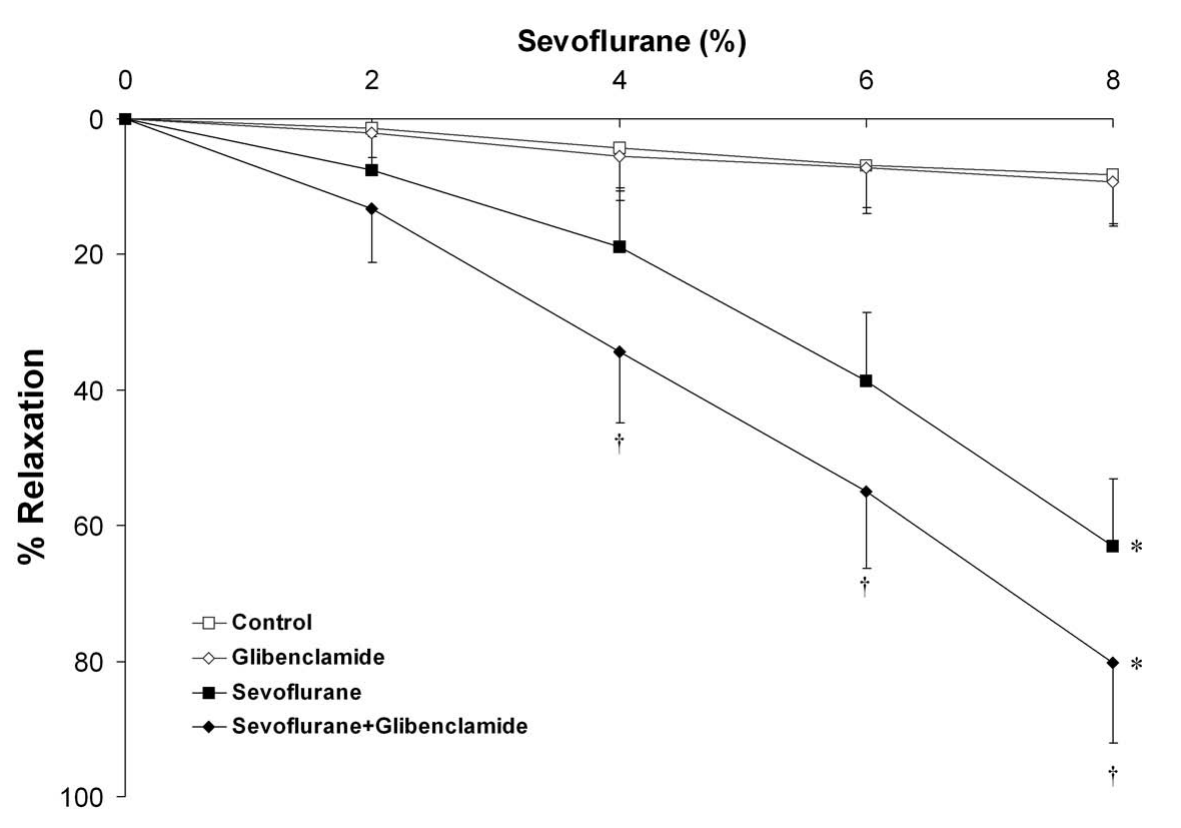
**Blockade of K_ATP _channels enhances the vasodilatory effect of sevoflurane**. Sevoflurane produced significant dose dependent vasodilation of chorionic plate arterial rings compared to control conditions. Prior blockade of K_ATP _channels by incubation in 1 × 10^-5 ^M glibenclamide (n = 10 rings per group) enhanced the vasodilatory effect of sevoflurane. *P < 0.05 compared to rings exposed to control conditions. (Two way RM-ANOVA). † P < 0.05 compared to rings exposed to sevoflurane plus vehicle at graded sevoflurane concentrations (post hoc between group t test).

In ***Series 2***, blockade of the ATP sensitive K^+ ^channel (K_ATP_) channel (n = 10 rings per group) with 1 × 10^-5 ^M glibenclamide significantly increased sevoflurane-mediated vasodilation compared to sevoflurane alone (Figure 5). Glibenclamide pre-treatment significantly decreased the EC_50r _for sevoflurane (5.5 ± 0.9% versus 6.8 ± 1.0%, P < 0.005).

### Sevoflurane and the Ca^++ ^Channel

**Figure 6:**
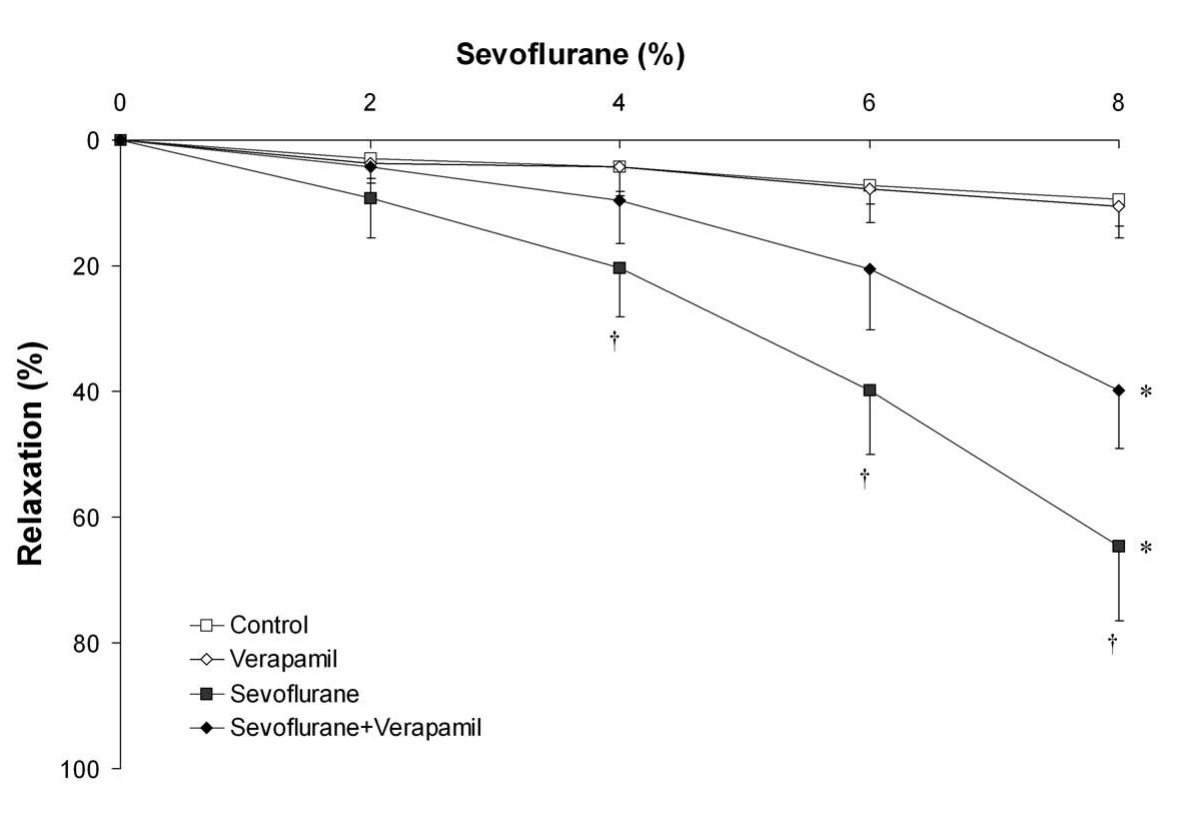
**Blockade of sarcolemmal voltage-operated Ca^++ ^channel (VOCC) attenuates the vasodilatory effect of sevoflurane**. Sevoflurane produced significant dose dependent vasodilation of chorionic plate arterial rings compared to control conditions. Prior blockade of VOCC's by incubation in 1 × 10^-6 ^M verapamil (n = 10 rings per group) reduced the vasodilatory effect of sevoflurane. *P < 0.05 compared to rings exposed to control conditions. (Two way RM-ANOVA). † P < 0.05 compared to rings exposed to sevoflurane plus verapamil at graded sevoflurane concentrations (post hoc between group t test).

**Figure 7:**
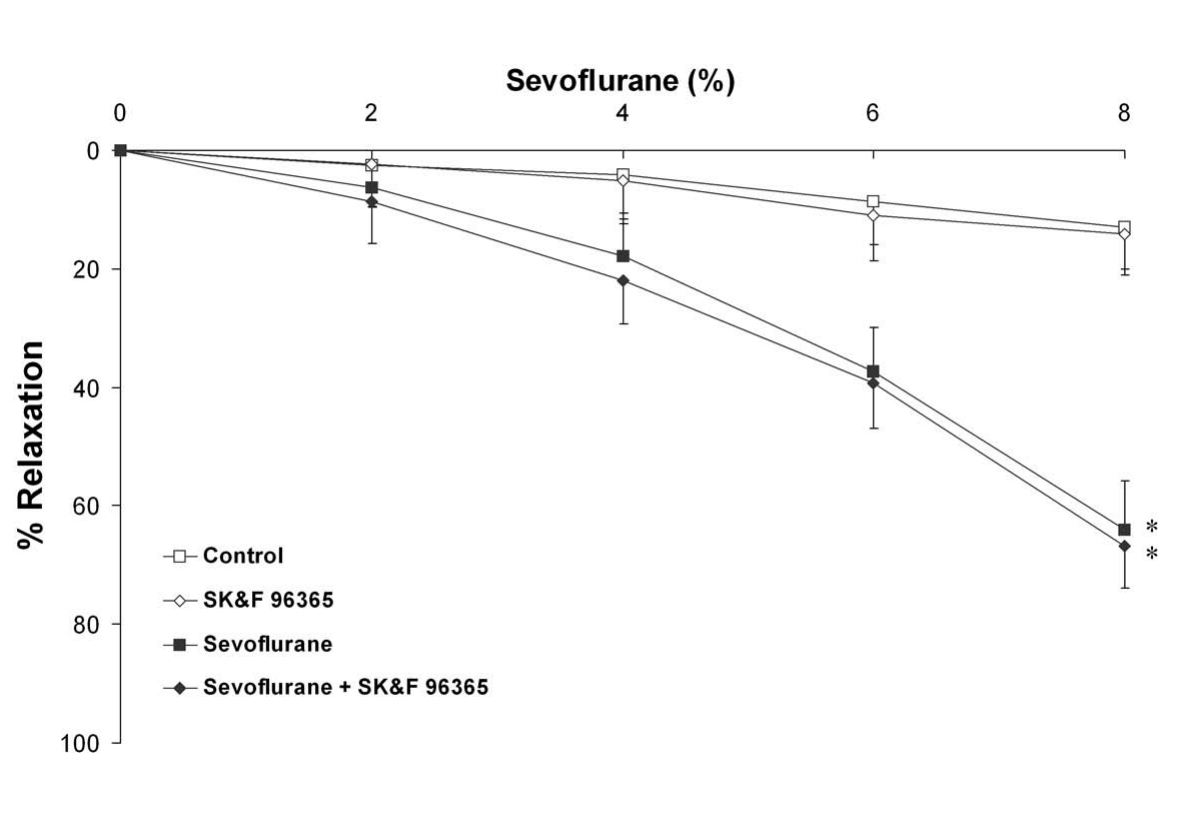
**Blockade of sarcolemmal voltage-independent Ca^++ ^channels (VICC), by prior incubation with 1 × 10^-4 ^M SK&F 96365 (n = 10 rings per group), does not alter sevoflurane mediated vasodilation**. Sevoflurane produced significant dose dependent vasodilation compared to control conditions, which was not modulated by SK&F 96365. *P < 0.05 compared to rings exposed to control conditions. (Two way RM-ANOVA).

In ***Series 3***, blockade of the sarcolemmal voltage-operated Ca^++ ^channel (n = 10 rings per group) with 1 × 10^-6 ^M verapamil significantly inhibited sevoflurane-mediated vasodilation compared to sevoflurane alone (Figure 6). Verapamil pre-treatment significantly increased the EC_50r _for sevoflurane (9.8 ± 1.1% versus 6.7 ± 1.0%, P < 0.01). In ***Series 4A***, blockade of the sarcolemmal voltage-independent Ca^++ ^channel with 3 × 10^-5 ^M SK&F 96365 (n = 10 rings per group) did not alter sevoflurane-mediated vasodilation (data not presented). In ***Series 4B***, higher concentrations (1 × 10^-4 ^M) of SK&F 96365 (n = 10 rings per group) were similarly ineffective in altering sevoflurane-mediated vasodilation. (Figure 7), and did not alter the EC_50r _for sevoflurane (6.7 ± 0.9% versus 6.9 ± 1.0%, P = 0.7).

**Figure 8:**
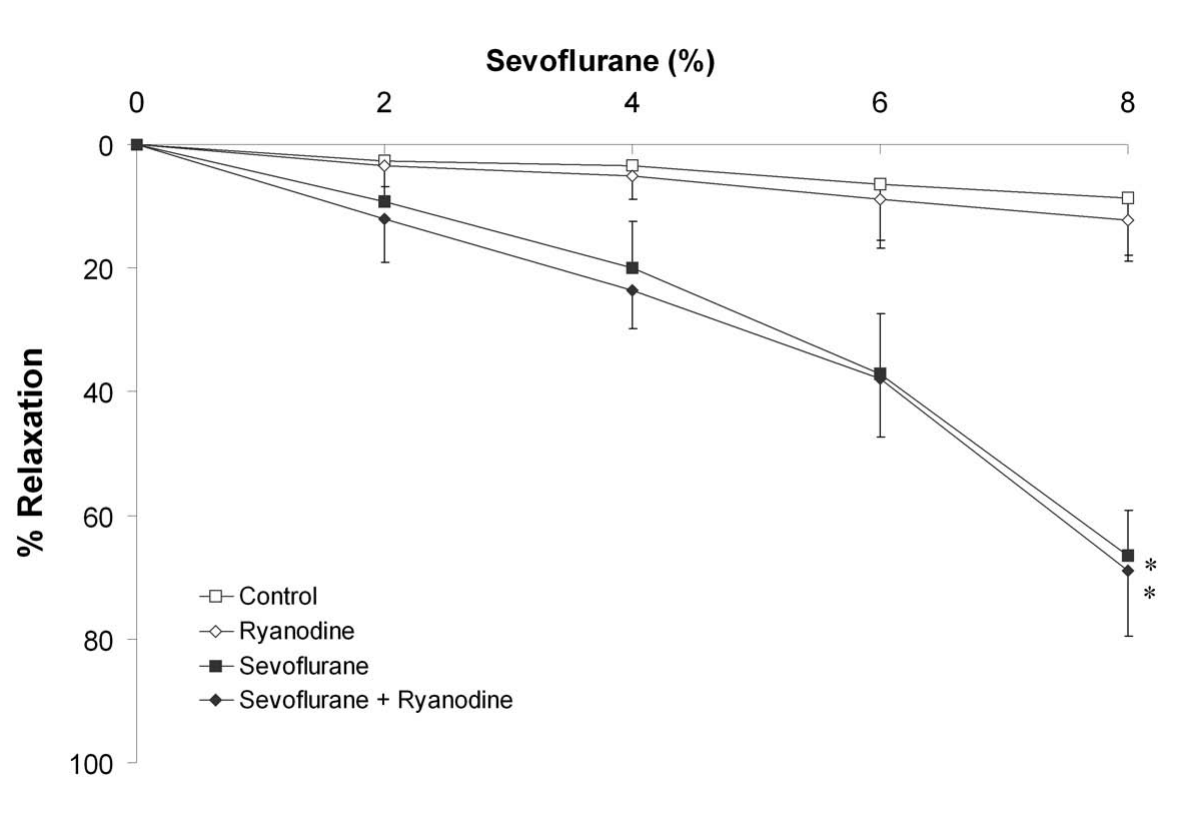
**Blockade of sarcoplasmic reticulum Ca^++ ^channel, by prior incubation with 5 × 10^-5 ^M ryanodine (n = 10 rings per group), does not alter sevoflurane mediated vasodilation**. Sevoflurane produced significant dose dependent vasodilation compared to control conditions, which was not modulated by ryanodine. *P < 0.05 compared to rings exposed to control conditions. (Two way RM-ANOVA).

In ***Series 5A***, blockade of the sarcoplasmic reticulum Ca^++ ^channel with 5 × 10^-6 ^M ryanodine (n = 10 rings per group) did not alter sevoflurane-mediated vasodilation (data not presented). In ***Series 5B***, higher concentrations (5 × 10^-5 ^M) of ryanodine (n = 10 rings per group) were similarly ineffective in altering sevoflurane-mediated vasodilation. (Figure 8), and did not alter the EC_50r _for sevoflurane (6.7 ± 1.0% versus 6.8 ± 0.9%, P = 0.8).

## Discussion

A substantial number of patients require general anaesthesia during pregnancy, for a variety of indications, including non-obstetric surgery [22]. In fact, the need for general anaesthesia during pregnancy may increase in future years, due to the emergence of *in utero *foetal surgical techniques, such as the *ex utero *intrapartum treatment (EXIT) procedure [23]. These procedures generally employ higher sevoflurane concentrations in order to produce uterine relaxation and facilitate *in utero *surgery, with concentrations of 4 – 5% sevoflurane reported for the EXIT procedure [24].

We have recently demonstrated that sevoflurane produces dose dependent vasodilation in the foeto-placental circulation *in vitro *[4]. These effects of sevoflurane are mediated *via *a mechanism that is NO-independent and which was mediated in part *via *the generation of lipoxygenase derived vasodilator prostanoids. However, blockade of the lipoxygenase enzyme does not abolish sevoflurane-mediated vasodilation, suggesting that other mechanisms of action may contribute to this effect. One potential mechanism by which sevoflurane may produce vasodilation in this circulation would be *via *the modulation of ion channel function. The effects of volatile anaesthetic agents on K^+ ^channels appear to be agent and vascular bed specific. Halothane-mediated vasodilation of conducting and resistance coronary arteries is mediated *via *activation of the adenosine triphosphate-sensitive potassium (K_ATP_) channels [5]. In contrast, sevoflurane increases coronary collateral blood flow *via *mechanisms that appear to be independent of K_ATP _channel activation [7], but which involve activation of K_Ca++ _channels [8]. These findings lead us to hypothesize that sevoflurane may produce vasodilation in the foeto-placental circulation *via *activation of K_Ca++ _and/or K_ATP _channels.

Our findings demonstrate that sevoflurane did not produce vasodilation *via *activation of either the K_Ca++ _or the K_ATP _channel. In fact, prior exposure of the chorionic arterial rings to TEA, which blocks the K_Ca++ _channels, and glibenclamide, which blocks the K_ATP _channel, clearly enhanced sevoflurane-mediated vasodilation. This suggests that sevoflurane in part inhibited or blocked these K^+ ^channels, contrary to our contention. We wished to confirm that sevoflurane did indeed block the K_Ca++ _and K_ATP _channels. If this contention were correct, then prior incubation of the rings with sevoflurane would prevent binding of agents that block these channels, and thereby abolish their effect. In further experiments, carried out to clarify this issue, blockade of K_Ca++ _channels by TEA caused transient vasoconstriction, which demonstrates a role for the K_Ca++ _channels in regulating vasomotor tone in the foeto-placental circulation. However, prior incubation of the rings with sevoflurane prior to exposure to TEA abolished this effect. Taken together, these findings indicate that sevoflurane blocks the K_Ca++ _channel. These findings are not inconsistent with previous literature. Halothane has been demonstrated to depress the function of a wide variety of both invertebrate and mammalian K^+ ^channels [25], including the K_Ca++ _channel in rodents [26], and in canine cerebral and coronary vascular smooth muscle [27]. Our findings emphasise the potential for volatile anaesthetic agent to exert effects on vascular bed specific the K_Ca++ _or the K_ATP _channel.

Our second hypothesis was that sevoflurane produced vasorelaxation in part *via *the blockade of calcium ion channels. We therefore examined the potential for the effects of sevoflurane to be mediated *via *the sarcolemmal voltage-operated Ca^++ ^channel (VOCC) [9], the sarcolemmal voltage-independent Ca^++ ^channels (VICC) [9,10], and/or the sarcoplasmic reticulum calcium channel. Our findings demonstrate that sevoflurane produced vasodilation, at least in part, *via *an effect on the sarcolemmal voltage-operated calcium channel (VOCC). Prior incubation with the VOCC channel blocker verapamil, at concentrations similar to that previously reported [17,18], significantly inhibited sevoflurane-mediated vasodilation of the pre-constricted chorionic plate arterial rings. These findings demonstrate that sevoflurane-mediated vasodilation in this circulation is dependent in part on a blocking effect at the VOCC channel. This effect of sevoflurane would reduce intracellular calcium influx, and thereby reduce chorionic plate arterial vasomotor tone. In contrast, the vasomotor effects of sevoflurane appear to be independent of both the sarcolemmal voltage-independent calcium channel and alterations in sarcoplasmic reticulum calcium release.

It is clear, based on these findings, and those of our prior study [4], that sevoflurane modulates vasomotor tone in the foeto-placental circulation *via *a number of separate and competing mechanisms. Sevoflurane inhibits the production of vasodilator prostanoids, and also inhibits the activity of the K_Ca++ _and K_ATP _channels, which would act to increase vascular tone. However, these effects are counterbalanced by sevoflurane-mediated inhibition of vasoconstrictor eicosanoid production, and blockade of Ca^++ ^channels, which act to produce vasodilation. The net effect of these contrasting mechanisms of action is to produce dose-dependent vasodilation. Of interest, halothane has been demonstrated to exert similar effects on ion channel function in isolated smooth muscle cells of canine cerebral arteries [28], and in canine coronary arterial cells [29]

There are some limitations to this study. Firstly, these studies are conducted in second order chorionic plate arteries. Characterisation of the effects of sevoflurane on smaller vessels in the foeto-placental circulation (i.e. placental resistance arteries) is also required. Secondly, chorionic plate arterial ring samples were obtained from healthy parturients. Characterisation of the effects of sevoflurane in the setting of compromised utero-placental circulation, in which foetal hypoxia may be more prevalent would add further useful information. Thirdly, SK&F 96365, which was utilised to determine the role of the voltage-independent Ca^++ ^channels in mediating the vasorelaxant effect of sevoflurane, may also have a weak VOCC blocking action [9,10]. However, these effects are only seen at higher concentrations that were used in these studies, and in any case SK&F 96365 did not modulate the effect of sevoflurane in these studies. Fourthly, our findings may not apply to other inhaled anaesthetic agents, especially in a quantitative manner. The effects of other volatile anaesthetics will need to be separately determined. Fifthly, these experiments were conducted under conditions of hyperoxia, in contrast to the hypoxic conditions normally experienced by the placental circulation. Hyperoxia may cause vasoconstriction in this circulation. However the oxygen levels used in these studies are widely used in isolated placental vessel preparations [30-32]. Perfusate oxygen tensions were the same across all experimental groups in this study. Therefore, the impact of the oxygen concentrations in these experiments is likely to have been limited. Finally, there are limitations in extrapolating from *in vitro *experiments to the *in vivo *situation.

## Conclusion

We conclude that sevoflurane dose dependently dilates the foeto-placental vasculature, by a mechanism mediated in part *via *blockade of the voltage-operated calcium channel, in this isolated placental vessel model. Further study is required to investigate determine the clinical significance of these findings.

## Abbreviations

ANOVA: analysis of variance; ATP: Adenosine triphosphate; Ca^++^: Calcium ion; CaCl_2_: Calcium chloride; EC_50r_: concentration required dilate rings by 50%; EXIT: *ex utero *intrapartum treatment procedure; HIV: Human Immunodeficiency virus; K^+^: Potassium ion; K_ATP_: ATP sensitive K^+ ^channel; K_Ca++_: calcium-activated K^+ ^channel; KCl: Potassium Chloride; kPa: kilopascals; M: molar; MgSO_4_; NaCl: Sodium Chloride; NaHCO_3_: sodium bicarbonate; Na_2_HPO_4_: sodium hydrogen phosphate; NO: nitric oxide; P_CO2_: Partial pressure of carbon dioxide; P_O2_: Partial pressure of oxygen; PSS: physiologic saline solution; Sevo: sevoflurane; SD: standard deviation; TEA: Tetraethylammonium; U46619: 9,11-dideoxy-11α, 9α-epoxymethanoprostagladin F_2α_; VICC: voltage-independent Ca^++ ^channels; VOCC: voltage-operated Ca^++ ^channel.

## Competing interests

The authors declare that they have no competing interests.

## Authors' contributions

CHM and BDH conceived of the study, and participated in its design and execution and helped to draft the manuscript. JJ, and RFF performed experiments, and helped to draft the manuscript. CDL determined concentrations of sevoflurane in the perfusate using gas chromatography-mass spectrometric analyses. NMF and JGL participated in the design and coordination of the study, performed the statistical analysis, and helped to draft the manuscript. All authors read and approved the final manuscript.

## Pre-publication history

The pre-publication history for this paper can be accessed here:


